# Knowledge, attitudes, and practices regarding antibiotic use in Bangladesh: Findings from a cross-sectional study

**DOI:** 10.1371/journal.pone.0297653

**Published:** 2024-02-12

**Authors:** Md. Abu Raihan, Md. Saiful Islam, Shariful Islam, A. F. M. Mahmudul Islam, Khandaker Tanveer Ahmed, Tania Ahmed, Md. Nahidul Islam, Shamsunnahar Ahmed, Mysha Samiha Chowdhury, Dipto Kumar Sarker, Anika Bushra Lamisa

**Affiliations:** 1 Department of Microbiology, Jahangirnagar University, Savar, Dhaka, Bangladesh; 2 Department of Public Health and Informatics, Jahangirnagar University, Savar, Dhaka, Bangladesh; 3 Centre for Advanced Research Excellence in Public Health, Savar, Dhaka, Bangladesh; 4 Department of Microbiology, Noakhali Science & Technology University, Noakhali, Bangladesh; 5 Department of Pharmacy, Gono Bishwabidyalay, Savar, Dhaka, Bangladesh; 6 Department of Statistics, Jahangirnagar University, Savar, Dhaka, Bangladesh; 7 Department of Biochemistry and Microbiology, School of Health and Life Science, North South University, Dhaka, Bangladesh; Universidad Privada San Juan Bautista, PERU

## Abstract

**Background:**

Escalating antibiotic resistance presents a notable worldwide dilemma, pointing a large involvement of general population. The objective of this study was to assess knowledge, attitudes, and practices regarding the utilization of antibiotics among Bangladeshi residents.

**Methods:**

A cross-sectional study, conducted from January 01 to April 25, 2022, included 1,947 Bangladeshi adults with a history of antibiotic use, via online surveys and face-to-face interviews using a pretested semi-structured questionnaire. Descriptive statistics, Chi-square tests, and multivariate linear regression models were employed.

**Results:**

Mean scores for knowledge, attitudes, and practices were 6.59±1.20, 8.34±1.19, and 12.74±2.59, with correct rates of 73.22%, 92.67%, and 57.91%. Positive predictors for knowledge included being unmarried (β = 0.10, p = 0.001), higher education (College: β = 0.09, p = 0.025; Bachelor: β = 0.22, p<0.001; Master or above: β = 0.14, p<0.001), various professions (student: β = 0.57, p<0.001; housewife: β = 0.33, p<0.001; employee: β = 0.53, p<0.001; businessman: β = 0.31, p<0.001; unemployed: β = 0.15, p<0.001), and residing in semi-urban (β = 0.32, p<0.001) or urban areas (β = 0.15, p<0.001). Positive predictors for attitudes included being married (β = 0.18, p<0.001), specific professions (student: β = 1.06, p<0.001; housewife: β = 0.33, p<0.001; employee: β = 0.86, p<0.001; businessman: β = 0.37, p<0.001; unemployed: β = 0.47, p<0.001), higher SES (Lower-middle: β = 0.22, p<0.001; Middle: β = 0.26, p<0.001), and residing in semi-urban areas (β = 0.18, p<0.001); negative predictors included higher education (College: β = -0.12, p = 0.001; Master or above: β = -0.09, p = 0.008) and being rich (β = -0.13, p<0.001). Positive predictors for practices included being married (β = 0.18, p<0.001), specific professions (student: β = 0.32, p<0.001; employee: β = 0.43, p<0.001; businessman: β = 10, p = 0.034; unemployed: β = 0.11, p = 0.009), and higher SES (Lower-middle: β = 0.14, p = 0.009; Middle: β = 0.38, p<0.001; Higher-middle: β = 0.15, p = 0.008); negative predictors included higher education (College: β = -0.21, p<0.001), being rich (β = -0.12, p<0.001), residing in semi-urban (β = -0.14, p<0.001) or urban areas (β = -0.16, p<0.001).

**Conclusions:**

Participants exhibited adequate knowledge and positive attitudes but lagged behind in proper practice of antibiotic use. Proper initiatives should be tailored to enhance prudent antibiotic use and mitigate the risk of antimicrobial resistance.

## Introduction

Since the discovery of antibiotics approximately eight decades ago, there has been a revolution in the management, treatment, and outcome of infectious diseases [1]. Therefore, antibiotics are one of the most commonly prescribed, sold, and used drugs in the world [2]. In a number of Developing nations such as Bangladesh, Pakistan, Cameroon and Uganda, antibiotics are available without a prescription [3–5], resulting in indiscriminate antibiotic use. Antibiotics are administered at incorrect doses, for incorrect indications, at incorrect dosing intervals, and for too long or insufficient duration [3, 5].

The inappropriate use of antibiotics may result from a complex interaction between multiple factors, including prescriber behaviors and knowledge, diagnostic uncertainty, patient demand, self-medication, and poor patient-prescriber interaction, as well as macro-level factors including sociocultural, economic, and health care regulatory policy [3, 6–8]. In addition, the knowledge, beliefs, and attitudes of patients, as well as their expectations and past experiences with antibiotics, have contributed to the spread and emergence of resistant microorganisms [9]. Increased self-medication could result in resource waste, pathogen resistance, adverse reactions, incorrect self-diagnosis, delays in seeking appropriate care, and the risk of drug dependence and abuse [10, 11]. The use of antibiotics without a doctor’s prescription is the leading cause of antibiotic resistance [12, 13]. Many factors contribute to high antibiotic consumption, including non-restrictive prescription counter sales, a lack of consistent antibiotic use policies, and patient exposure to increasingly resistant microorganisms [14, 15]. Poor knowledge and less awareness are risk factors that contribute to the irrational use of antibiotics [16]. Antibiotic resistance develops due to a variety of factors, including the use of antibiotics that were previously prescribed, obtained from relatives or friends, or obtained without a prescription [17]. In Developing countries, Antibiotic resistance is responsible for significant mortality, morbidity, and costs [18]. Antibiotic consumption increased by 65 percent between 2000 and 2015 globally [19]. If nothing is done, antimicrobial resistance will endanger approximately 10 million lives per year, with a global economic output of 100 trillion USD, by the year 2050 [20, 21]. One of the most important aspects of controlling antibiotic resistance is changing the behavior of antibiotic consumers and providers [1]. As a result, major resistance control strategies advocate public education to promote appropriate antibiotic use [22, 23]. A positive attitude, adequate knowledge, and best practices regarding antibiotics can all play an important role in the rational use of antibiotics [24].

Several studies in Bangladesh have already confirmed that a considerable proportion of bacterial species especially *S*. *typhi*, *H*. *pylori*, and *E*. *coli* were resistant to several broad-spectrum antibiotics [25–28]. In other studies, it was found that multi-drug resistant *S*. *typhi* have become a major problem globally and have been reported not only in Latin America, Egypt, Nigeria, China, Korea, Vietnam and Philippines but also in South East Asia [29, 30]. Another study also demonstrated that two-thirds of the patients were resistant to meropenem, quinolones, and the piperacillin-tazobactam combination like broad spectrum antibiotics [31]. Therefore, the present scenario reiterates to the establishment of good practice of antibiotics in any setting.

Several studies were conducted on Knowledge, attitudes and practices regarding antibiotic use earlier in Bangladesh. But most of them were limited to specific area and specific population group such as public university students [32], or veterinary students [33], or patients of OPD (Outpatient Department) of a Dental College Hospital [34], or commercial poultry farmers [35]. For assessing an overall concept, limited population group is not enough and larger population group consisting with general public need to be included for assessing knowledge, attitudes and practices.

This study aimed to determine the general public’s knowledge, attitudes, and practices regarding antibiotic use in Bangladesh, which can aid in the planning and development of an effective and tailored educational intervention for them to combat the spread of antibiotic misuse and its consequences.

## Materials and method

### Study design and setting

A cross-sectional study was conducted in 37 districts (the administrative regions–Bandarban, Barishal, Bogura, Brahmanbaria, Chandpur, Chattogram, Cox’s Bazar, Cumilla, Dhaka, Dinajpur, Feni, Gaibandha, Gazipur, Gopalganj, Jamalpur, Jashore, Kurigram, Khulna, Manikganj, Munshiganj, Mymensingh, Narail, Narayanganj, Natore, Noakhali, Pirojpur, Rajshahi, Rangpur, Rangamati, Shariatpur, Shatkhira, Sherpur, Sirajganj, Sylhet, Sunamganj, Tangail, and Thakurgaon) of Bangladesh from January 01 to April 25, 2022. A total of 1,947 data were collected using a semi-structured questionnaire through online surveys (n = 1,163) and face-to-face interviews (n = 784) to ensure the enrollment of the participants those who did not use any online devices. In the online survey, we employed convenience sampling, leveraging Facebook, WhatsApp, and E-mail with Google Forms as our data collection platform. The survey link was disseminated to each participant via Facebook Messenger, WhatsApp, or Email. Face-to-face surveys utilized a purposive sampling approach, meticulously selecting participants based on predefined criteria aligned with research objectives. Data collection took place in a variety of settings, including community centers, workplaces, and public events, carefully chosen to match the study’s nature and the intended target population.

### Inclusion and exclusion criteria

Inclusion criteria of the participants included being adult (≥18 years), having antibiotics for themselves & family 90 days prior to the study, healthy (capable to participate in the study) & mentally stable (who had no evidence of mental illness), and having willingness to take part in the study. People who were sick or injured and mentally unstable, not having antibiotics for themselves or family, and having unwillingness were excluded from the study.

### Sampling technique and sample size

The sample size was determined using the Raosoft sample size calculator [36] using a margin of error of 5%, a confidence interval of 95%, a population size of 166,303,498 people, and an expected response of 50%. The minimum sample size estimated for the study was 385. In order to increase the statistical power of the study, a larger sample size of 1947 people (five times of estimation) were enrolled in the study.

### Ethics

This study was approved by the ethical review board of the Gono Bishwabidyalay, Savar, Dhaka, Bangladesh [Ref. No.: CMR/E/002]. Both verbal and written Informed consent was obtained from each eligible individual prior to enrolment in this study. An ICF (Informed Consent Form) was provided them who participated this study through online and filled up ICF was taken from them through online. Verbal consent was also taken from them. Those who participated this study face-to-face were given the hard copy of ICF form and written consent was taken from them and if anyone was unable to understand the ICF, the ICF was read through both in English and native language (Bangla) for better understanding for them and filled up forms along with their sign were taken according their consent. All data were collected anonymously and the privacy and confidentiality of participants’ information were strictly maintained.

### Study instrument

A semi-structured questionnaire was designed (S1 File) both in English and native Bangla language together for better understanding for all participants. The questionnaire was designed to get an overview of knowledge, attitudes & practices towards antibiotics uses among mass population. The majority of the questionnaire’s questions were culled from an existing study [37, 38]. A small-scale pilot study was conducted by a primary questionnaire among 98 people who had used antibiotics for themselves or a family member in the previous 90 days prior to the main survey to validate the suitability of the study instruments. Experts in the fields of statistics and epidemiology were consulted in order to refine and perfect the questionnaire based on the pilot study’s findings and questionnaire was modified.

### Measures

The final version of the questionnaire consisted of 38 questions broken down into four groups: demographic data [previously used in several studies [39–44], knowledge of antibiotic use [previously used in several studies [37, 38], attitudes towards antibiotic use [previously used in several studies [37, 38], and practices of antibiotic use [previously used in several studies [37, 38]. When comparing the overall results of each partition, each and every question is relevant.

### Demographic data

Demographic data consists of information on age, district, gender (male/ female/ prefer not to say), marital status (unmarried/ married/ divorced or divorcee/ widow or widower), living status (town or city/ sub-town/ village/ slum), education level (school or below/ college/ bachelor/ masters or above), profession (student/ housewife/ employee/ businessman/ day laborer/ unemployed) and economic status (rich/ higher middle class/ middle class/ lower middle class/ poor).

### Knowledge of antibiotic use

The section on knowledge of antibiotic use consisted of nine questions addressing normal flora of the body, concepts of drug sensitivity and susceptibility, the relationship between disease, drug resistance, and side effects of antibiotics, and perspectives on the efficacy of antibiotics. All the questions had dichotomous responses (i.e., Yes/ No).

### Attitudes towards antibiotic use

Nine questions were included in the section of attitudes towards antibiotic use about the severity of antibiotic abuse, its impact on the individual and his or her family, the reasons of antibiotic abuse, and the necessary procedures to end antibiotic abuse. All the questions had dichotomous responses (i.e., Agree/ Disagree).

### Practices of antibiotic use

The practice section consisted of 11 questions ranging from the frequency of antibiotic use in fever, infections, and other symptoms to drug withdrawal state. The responses consisted of “Always/ Sometimes/ Never”.

The categorization of knowledge, attitudes, and practices (KAP) concerning antibiotic use in our study was established by systematically evaluating respondents’ answers to specific questions customized for each category. For knowledge assessment, a set of nine questions probed participants’ comprehension of critical concepts, such as normal body flora, drug sensitivity, susceptibility, disease-drug relationships, antibiotic side effects, and perceptions of antibiotic efficacy. When participants provided correct answers, it signified that they possessed sound knowledge in the respective category. In the attitudes section, we included nine questions aimed at gauging participants’ perspectives on antibiotic use, covering topics like the severity of antibiotic abuse, its impact on individuals and families, reasons behind misuse, and measures to combat abuse. Responses in agreement with established guidelines or expert consensus were considered indicative of positive attitudes. Lastly, the practices assessment encompassed 11 questions evaluating participants’ behaviors and habits related to antibiotic use, including the frequency of use for various conditions and practices concerning drug withdrawal. When participants consistently selected responses aligned with recommended practices, it demonstrated desirable habits in the antibiotic use category. This structured approach allowed us to objectively categorize participants’ KAP, providing valuable insights into their antibiotic-related knowledge, attitudes, and practices.

### Study procedure

The study instrument (S1 File) as a google form was provided to the participants through online for those who participated through online. Then the instrument was filled up properly by themselves after taking their consent. As a means, invitations were dispatched to each participant once through a single platform, unlike Facebook Messenger, WhatsApp, or Email. To deter respondents from submitting duplicates, a restriction limiting the number of responses from a single IP address was implemented. Moreover, to ensure that multiple responses were restricted, a predetermined and unique code was allocated to each participant. Our distinctive code was partitioned into three distinct segments. The first segment consisted of four numeric digits, while the second segment incorporated three alphabets (KAP). In the subsequent third segment, the numeric digits from the initial segment were reversed. For face-to-face participants, study instrument (S1 File) was provided and read aloud both in English and native language (Bangla) for better understanding after taking their consent. After obtaining data from the participants (both from online & face-to-face interview), dataset was prepared in Microsoft Excel (Making anonymous) for further statistical analysis.

### Statistical analysis

All types of analysis were performed using the two statistical packages of software (SPSS Statistics version 25, and STATA version 13). For categorical variables, frequencies, and percentages were computed; means and standard deviations were reported for continuous variables. In addition, Chi-square tests were performed using the SPSS. Finally, the multivariate linear regression models were run separately using STATA including the significant variables of bivariate regression analysis to explore the correlates of knowledge, attitudes, and practices towards the antibiotics. The response variables for the multivariate linear regression analyses were knowledge score, attitudes score, and practices score, representing participants’ levels of knowledge, attitudes, and practices towards antibiotic use, respectively. Assumptions for the multivariate linear regression were rigorously validated. Linearity, independence, homoscedasticity, and normality of residuals were confirmed. Multicollinearity and perfect collinearity were addressed through VIF checks, ensuring VIF scores were less than 10. A *p*-value less than 0.05 was considered as statistically significant.

## Results

### General characteristics of participants

A total of 1947 participants (mean age: 26.25 ± 6.55 years; age range: 18–56 years) were included in the final analysis. Of them, more than half were males (57.11%), and unmarried (63.94%). Majority had bachelor level of education (55.78%), were students (56.24%), had middle SES (58.65%), and resided in urban areas (65.59%) (**Table 1**).

**Table 1:** General characteristics of the participants (N = 1947).

Variables	n (%)
**Age** (Mean ± SD)	26.25 ± 6.55
**Gender**	
Male	1112 (57.11)
Female	835 (42.89)
**Marital status**	
Unmarried	1245 (63.94)
Married	696 (35.75)
Divorced	6 (0.31)
**Education**	
School or below	144 (7.4)
College	452 (23.22)
Bachelor	1086 (55.78)
Master or above	265 (13.61)
**Profession**	
Student	1095 (56.24)
Housewife	60 (3.08)
Employee	494 (25.37)
Businessman	145 (7.45)
Day laborer	43 (2.21)
Unemployed	110 (5.65)
**Socioeconomic status**	
Poor	54 (2.77)
Lower-middle class	316 (16.23)
Middle class	1142 (58.65)
Higher-middle class	369 (18.95)
Rich	66 (3.39)
**Residence**	
Rural	303 (15.56)
Semi-urban	367 (18.85)
Urban	1277 (65.59)

A comparative analysis using a chi-square test was conducted to explore the general characteristics of online and offline participants. Specific variables—age interval (in 5-year), gender, marital status, education level, profession, socio-economic status, and residential location—were purposefully selected to enable a comprehensive comparative analysis of online and offline participants. These variables were chosen based on their theoretical relevance and empirical significance to our research inquiry, as they can potentially serve as determinants of participant behavior in both online and offline settings. For instance, age intervals were chosen to capture age-related variations in participation patterns, while gender was included due to its established impact on internet usage behavior. Through the deliberate selection of participants from both contexts, an effective comparison and clarification of the factors influencing participant behavior are facilitated. It should be recognized that respondents from specific disciplines, such as those with backgrounds in medical and allied sciences, may already possess prior knowledge and attitudes regarding antibiotics. The potential bias stemming from this inherent familiarity will be duly acknowledged and thoughtfully taken into account during our analysis. The results revealed no significant difference between the two groups, as all comparative variables had p-values less than 0.05. These findings suggest that participants from both settings shared similar traits and behaviors (**Table 2**).

**Table 2:** Comparison of general characteristics of the online and offline participants.

Variables	Overall	Online	Offline	*p*-value
	n (%)	n (%)	n (%)	
**Age Interval (5 years)**				
< 20 years	229 (11.76)	151 (12.98)	78 (9.95)	<0.001
21–25 years	1002 (51.46)	600 (51.59)	402 (51.28)	
26–30 years	413 (21.21)	242 (20.81)	171 (21.81)	
31–35 years	146 (7.50)	62 (5.33)	84 (10.71)	
36–40 years	36 (1.85)	18 (1.55)	18 (2.30)	
41–45 years	79 (4.06)	66 (5.67)	13 (1.66)	
46–50 years	18 (0.92)	6 (0.52)	12 (1.53)	
51–55 years	18 (0.92)	12 (1.03)	6 (0.77)	
56–60 years	6 (0.31)	6 (0.52)	0 (0)	
**Gender**				
Male	1112 (57.11)	651 (55.98)	461 (58.80)	0.027
Female	835 (42.89)	512 (44.02)	323 (41.20)	
**Marital Status**				
Unmarried	1245 (63.94)	787 (67.67)	458 (58.42)	<0.001
Married	696 (35.75)	376 (32.33)	320 (40.82)	
Divorced	6 (0.31)	0 (0)	6 (0.77)	
**Education**				
School or below	144 (7.40)	1 (0.09)	143 (18.24)	<0.001
College	452 (23.22)	284 (24.42)	168 (21.43)	
Bachelor	1086 (55.78)	694 (59.67)	392 (50)	
Masters or above	265 (13.61)	184 (15.82)	81 (10.33)	
**Profession**				
Unemployed	110 (5.65)	26 (2.24)	84 (10.71)	<0.001
Housewife	60 (3.08)	42 (3.61)	18 (2.30)	
Student	1095 (56.24)	699 (60.10)	396 (50.51)	
Day Laborer	43 (2.21)	31 (2.67)	12 (1.53)	
Employee	494 (25.37)	332 (28.55)	162 (20.66)	
Businessman	145 (7.45)	33 (2.84)	112 (14.29)	
**Socio-economic Status**				
Poor	54 (2.77)	43 (3.70)	11 (1.40)	<0.001
Lower-middle class	316 (16.23)	208 (17.88)	108 (13.78)	
Middle class	1142 (58.65)	645 (55.46)	497 (63.39)	
Higher middle class	369 (18.95)	201 (17.28)	168 (21.43)	
Rich	66 (3.39)	66 (5.67)	0 (0)	
**Residence**				
Urban	1277 (65.59)	791 (68.01)	486 (61.99)	0.022
Semi-urban	367 (18.85)	206 (17.71)	161 (20.54)	
Rural	303 (15.56)	166 (14.27)	137 (17.47)	

### Knowledge towards antibiotics

**Fig 1:**
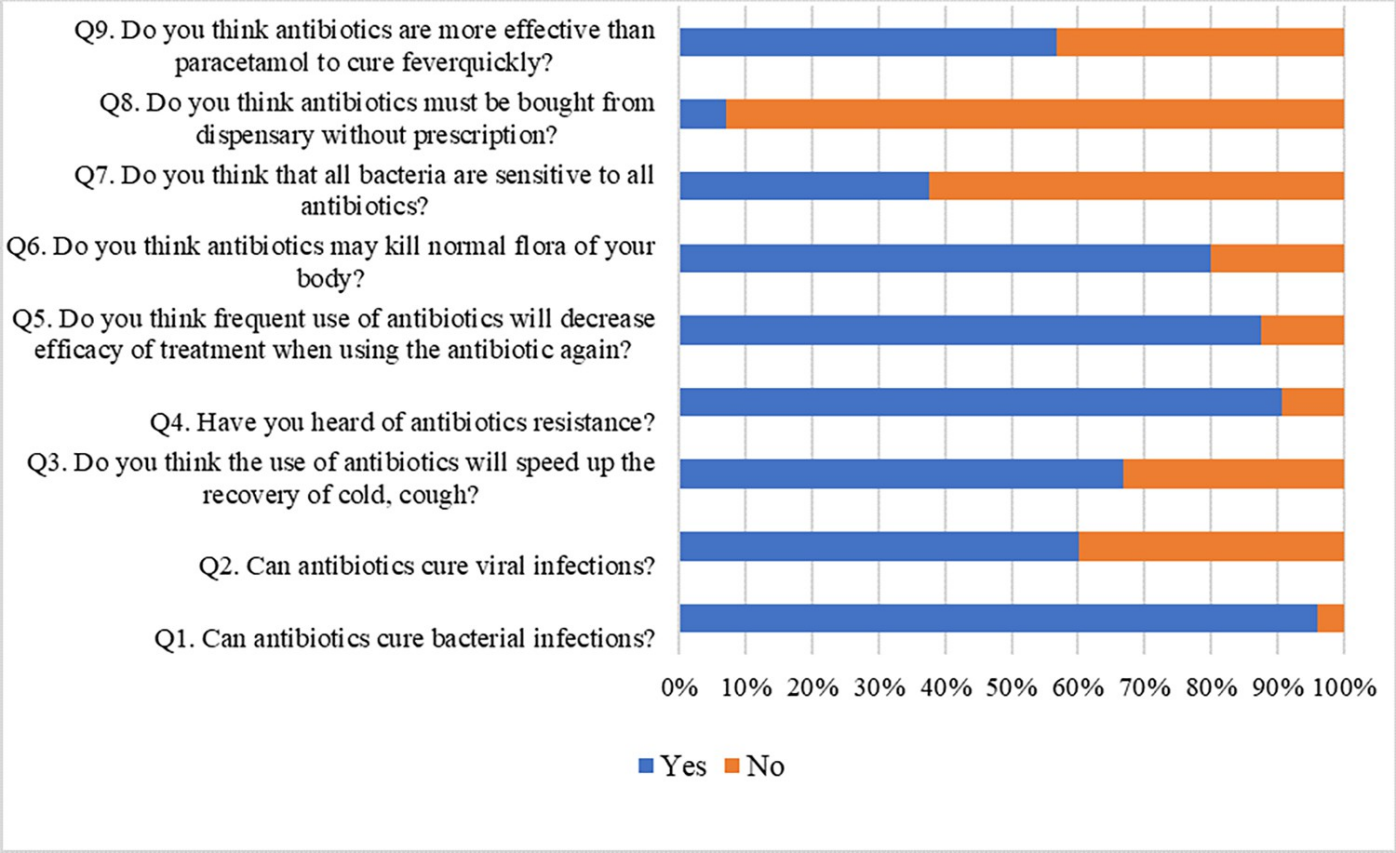


The responses of knowledge items were presented in the **Fig 1**. The mean score of knowledge was 6.59 (SD = 1.20) out of 9, indicating an overall correct rate of 73.22%.

As per as multiple linear regression analysis, the positively predicting factors of knowledge score included: ⅰ) being unmarried (β = 0.10, *p* = 0.001), ⅱ) having higher education (for college [β = 0.09, *p* = 0.025], for bachelor [β = 0.22, *p* < 0.001], and for ‘master or above’ [β = 0.14, *p* < 0.001] in reference to ‘school or below’), ⅲ) profession (for student [β = 0.57, *p* < 0.001], for housewife [β = 0.33, *p* < 0.001], for employee [β = 0.53, *p* < 0.001], for businessman [β = 0.31, *p* < 0.001], for unemployed [β = 0.15, *p* < 0.001] in reference to day laborer), and ⅳ) living in semi-urban (β = 0.32, *p* < 0.001) and urban areas (β = 0.15, *p* < 0.001) in reference to rural areas (**Table 3**). The regression model produced an adjusted R-squared of 0.357.

**Table 3:** Regression analysis by knowledge towards antibiotics.

Variables	Overall	*Bivariate regression analysis*					*Multiple regression analysis*				
	Mean (SD)	B	SE	T	β	*p*-value	B	SE	t	β	*p*-value
**Age**	6.59 (1.20)	0.02	0.00	3.54	0.08	<0.001	0.01	0.01	1.51	0.05	0.131
**Gender**											
Female	6.6 (1.14)	0.01	0.06	0.18	<0.01	0.859	⸻	⸻	⸻	⸻	⸻
Male	6.59 (1.24)	Ref.									
**Marital status**											
Unmarried	6.63 (1.23)	0.14	0.06	2.39	0.05	0.017	0.25	0.07	3.40	0.10	**0.001**
Divorced	8 (0)	1.50	0.49	3.06	0.07	0.002	0.82	0.47	1.76	0.04	0.079
Married	6.5 (1.13)	Ref.					Ref.				
**Education**											
College	6.42 (1.18)	0.04	0.11	0.36	0.01	0.721	0.25	0.11	2.25	0.09	**0.025**
Bachelor	6.66 (1.18)	0.29	0.11	2.72	0.12	0.006	0.55	0.10	5.32	0.22	**<0.001**
Master or above	6.7 (1.24)	0.32	0.12	2.61	0.09	0.009	0.50	0.12	4.15	0.14	**<0.001**
School or below	6.38 (1.26)	Ref.					Ref.				
**Profession**											
Student	6.6 (1.15)	1.60	0.18	8.88	0.66	<0.001	1.38	0.19	7.29	0.57	**<0.001**
Housewife	7.5 (0.68)	2.50	0.23	10.79	0.36	<0.001	2.32	0.25	9.37	0.33	**<0.001**
Employee	6.69 (1.26)	1.69	0.18	9.18	0.61	<0.001	1.47	0.20	7.55	0.53	**<0.001**
Businessman	6.67 (0.94)	1.67	0.20	8.29	36.00	<0.001	1.43	0.21	6.73	0.31	**<0.001**
Unemployed	6.05 (1.4)	1.05	0.21	5.01	0.20	<0.001	0.77	0.22	3.51	0.15	**<0.001**
Day laborer	5 (0)	Ref.					Ref.				
**Socioeconomic status**											
Lower-middle class	6.36 (1.29)	-0.09	0.18	-0.49	-0.03	0.621	⸻	⸻	⸻	⸻	⸻
Middle class	6.71 (1.23)	0.27	0.17	1.59	0.11	0.111	⸻	⸻	⸻	⸻	⸻
Higher-middle class	6.45 (1.02)	0.00	0.17	0.02	<0.01	0.988	⸻	⸻	⸻	⸻	⸻
Rich	6.55 (0.9)	0.10	0.22	0.46	0.02	0.645	⸻	⸻	⸻	⸻	⸻
Poor	6.44 (1.27)	Ref.									
**Residence**											
Semi-urban	7.11 (1.06)	1.22	0.09	13.70	0.40	<0.001	0.98	0.09	10.56	0.32	**<0.001**
Urban	6.6 (1.12)	0.71	0.07	9.67	0.28	<0.001	0.37	0.08	4.70	0.15	**<0.001**
Rural	5.89 (1.36)	Ref.					Ref.				

### Attitudes towards antibiotics

**Fig 2:**
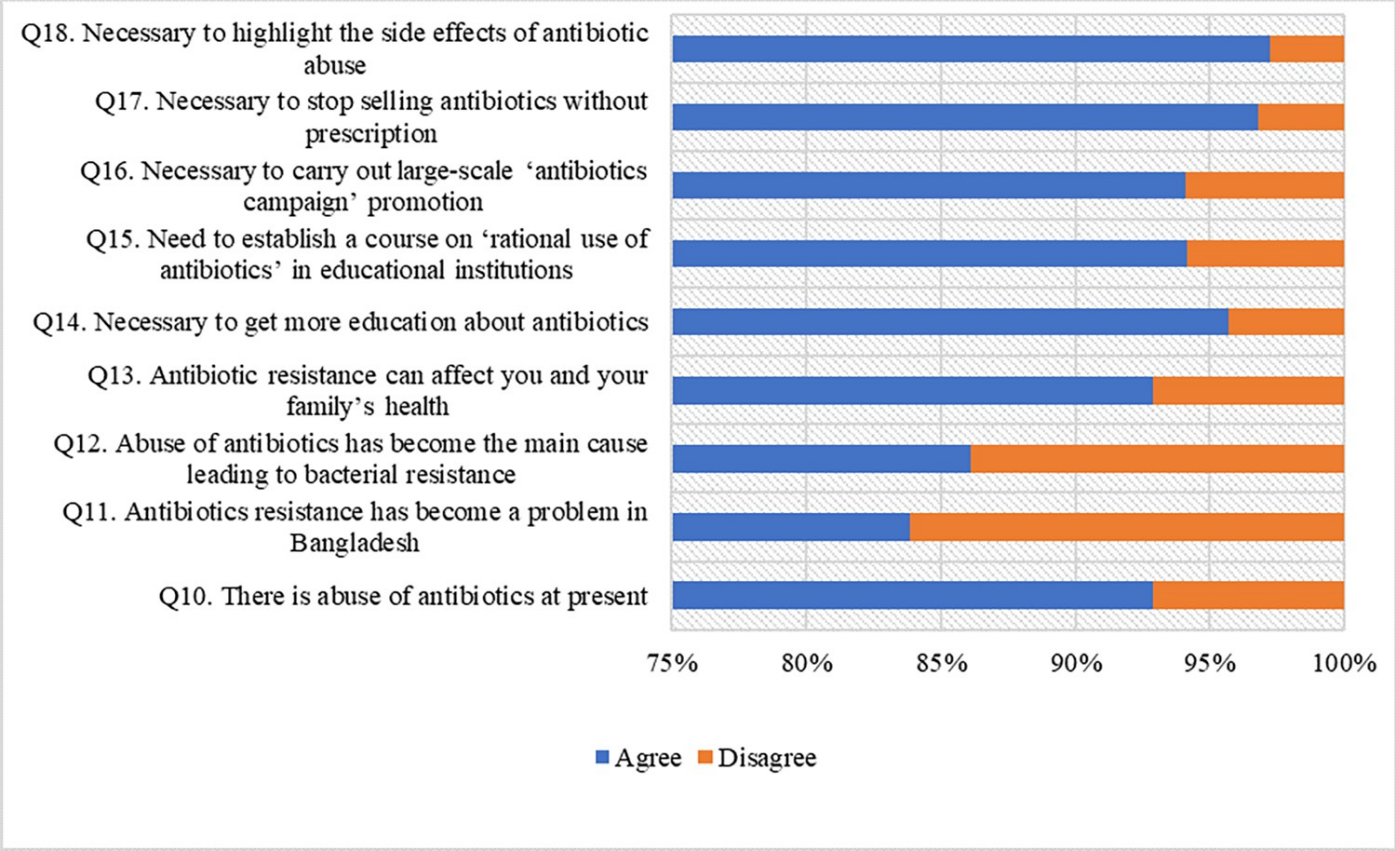


The responses of attitudes items are presented in **Fig 2**. The mean score of attitudes was 8.34 (SD = 1.19) out of 9, indicating an overall correct rate of 92.67%.

In the multiple linear regression analysis, the positively predicting factors of attitudes score included: ⅰ) being married (β = 0.18, *p* < 0.001), ⅱ) profession (for student [β = 1.06, *p* < 0.001], for housewife [β = 0.33, *p* < 0.001], for employee [β = 0.86, *p* < 0.001], for businessman [β = 0.37, *p* < 0.001], and for unemployed [β = 0.47, *p* < 0.001] in reference to day laborer), ⅲ) SES (for lower-middle SES [β = 0.22, *p* < 0.001], and for middle SES (β = 0.26, *p* < 0.001] in reference to poor), and ⅳ) living in semi-urban (β = 0.18, *p* < 0.001) in reference to rural areas (**Table 4**). Whereas, the negatively predicting factors of attitudes score were: ⅰ) education (for college [β = -0.12, *p* = 0.001], and for ‘master or above’ [β = -0.09, *p* = 0.008] in reference to ‘school or bellow’), and ⅱ) being rich (β = -0.13, *p* < 0.001) in reference to poor SES (**Table 4**). The regression model yielded an adjusted R-squared of 0.327.

**Table 4:** Regression analysis by attitudes towards antibiotics.

Variables	Overall	*Bivariate regression analysis*					*Multiple regression analysis*				
	Mean (SD)	B	SE	T	β	*p*-value	B	SE	T	β	*p*-value
**Age**	8.34 (1.19)	0.01	0.00	1.80	0.04	0.071	⸻	⸻	⸻	⸻	⸻
**Gender**											
Female	8.49 (1)	0.26	0.05	4.89	0.11	<0.001	0.01	0.06	0.09	<0.01	0.928
Male	8.22 (1.3)	Ref.					Ref.				
**Marital status**											
Married	8.53 (0.91)	0.31	0.06	5.52	0.12	<0.001	0.46	0.06	7.37	0.18	**<0.001**
Divorced	9 (0)	0.77	0.48	1.61	0.04	0.108	0.17	0.43	0.40	0.01	0.690
Unmarried	8.23 (1.3)	Ref.					Ref.				
**Education**											
College	8.16 (1.29)	-0.38	0.11	-3.35	-0.13	0.001	-0.33	0.10	-3.24	-0.12	**0.001**
Bachelor	8.42 (1.17)	-0.13	0.11	-1.21	-0.05	0.227	0.03	0.10	0.31	0.01	0.760
Master or above	8.21 (1.22)	-0.33	0.12	-2.74	-0.10	0.006	-0.30	0.11	-2.67	-0.09	**0.008**
School or below	8.54 (0.77)	Ref.					Ref.				
**Profession**											
Student	8.4 (1.2)	2.40	0.17	13.87	1.00	<0.001	2.54	0.18	14.02	1.06	**<0.001**
Housewife	8.8 (0.61)	2.80	0.22	12.61	0.41	<0.001	2.26	0.24	9.51	0.33	**<0.001**
Employee	8.4 (0.99)	2.40	0.18	13.60	0.88	<0.001	2.34	0.19	12.38	0.86	**<0.001**
Businessman	7.76 (1.48)	1.76	0.19	9.11	0.39	<0.001	1.69	0.21	8.11	0.37	**<0.001**
Unemployed	8.89 (0.31)	2.89	0.20	14.46	0.56	<0.001	2.42	0.21	11.75	0.47	**<0.001**
Day laborer	6 (0)	Ref.					Ref.				
**Socioeconomic status**											
Lower-middle class	8.34 (1.04)	0.56	0.17	3.38	0.18	0.001	0.69	0.17	4.20	0.22	**<0.001**
Middle class	8.55 (0.9)	0.77	0.16	4.87	0.32	<0.001	0.62	0.15	3.99	0.26	**<0.001**
Higher-middle class	8.02 (1.53)	0.25	0.17	1.49	0.08	0.136	0.17	0.16	1.06	0.06	0.290
Rich	6.91 (2.37)	-0.87	0.21	-4.18	-0.13	<0.001	-0.83	0.20	-4.27	-0.13	**<0.001**
Poor	7.78 (0.42)	Ref.					Ref.				
**Residence**											
Semi-urban	8.84 (0.58)	0.82	0.09	9.09	0.27	<0.001	0.56	0.09	6.52	0.18	**<0.001**
Urban	8.27 (1.22)	0.25	0.07	3.38	0.10	0.001	0.09	0.08	1.18	0.04	0.238
Rural	8.02 (1.38)	Ref.					Ref.				

### Practices towards antibiotics

**Fig 3:**
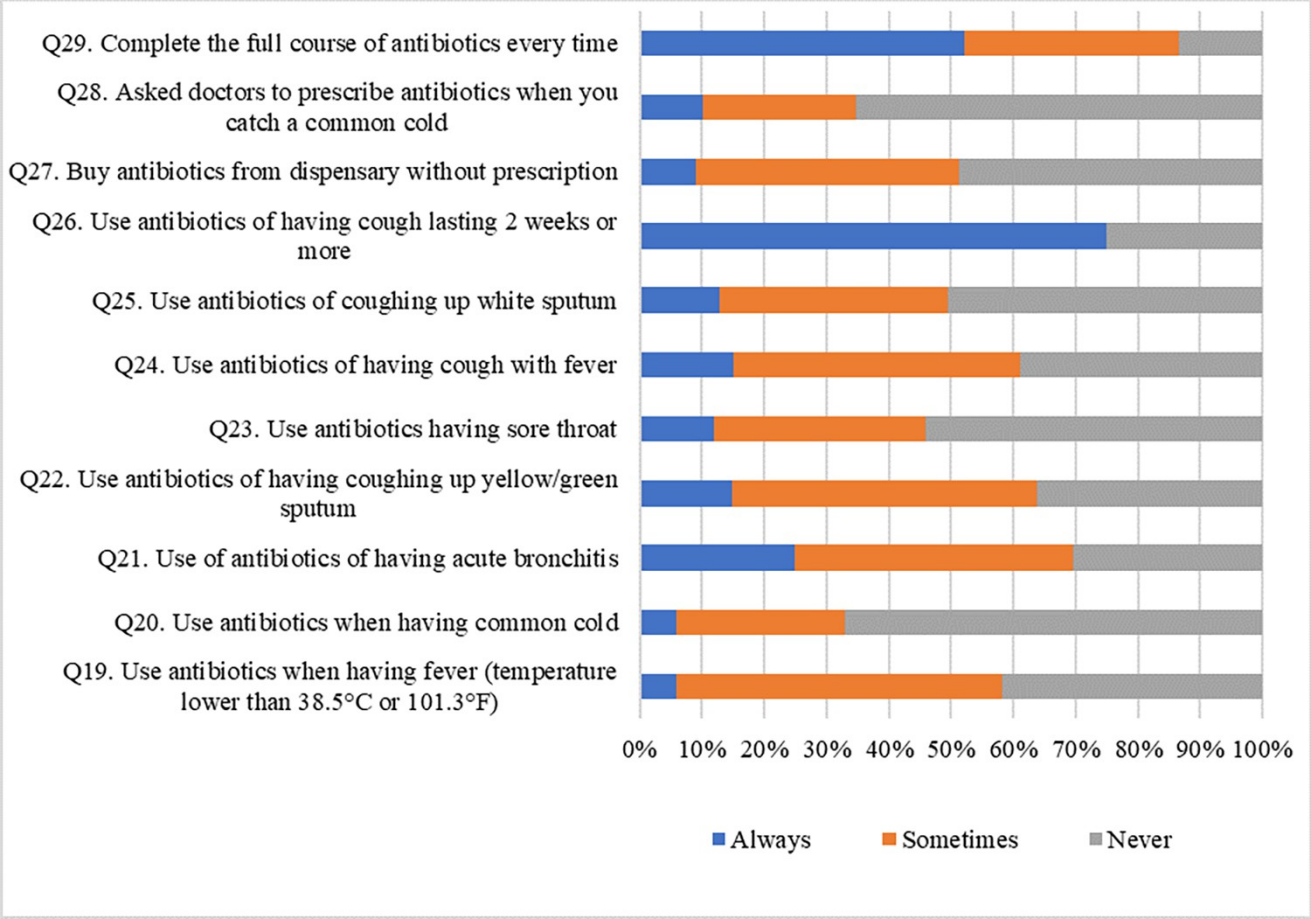


The responses of practices items are presented in **Fig 3**. The mean score of practices was 12.74 (SD = 2.59) out of 22, indicating an overall correct rate of 57.91%.

In the multiple regression analysis, the positively predicting factors of practices score included: ⅰ) being married (β = 0.18, *p* < 0.001), ⅱ) profession (for student [β = 0.32, *p* < 0.001], for employee [β = 0.43, *p* < 0.001], for businessman [β = 10, *p* = 0.034], and for unemployed [β = 0.11, *p* = 0.009] in reference to day laborer), and ⅲ) SES (for lower-middle SES [β = 0.14, *p* = 0.009], for middle SES [β = 0.38, *p* < 0.001], and for higher-middle SES [β = 0.15, *p* = 0.008] in reference to poor) (**Table 5**). Whereas, the negatively predicting factors of attitudes score were: ⅰ) education (for college [β = -0.21, *p* < 0.001] in reference to ‘school or bellow’), and ⅱ) being rich (β = -0.12, *p* < 0.001) in reference to poor SES, and ⅳ) living in semi-urban (β = -0.14, *p* < 0.001), and urban (β = -0.16, *p* < 0.001) areas in reference to rural areas (**Table 5**). The adjusted R-squared was 0.377 for the regression model.

**Table 5:** Regression analysis by practices towards antibiotics.

Variables	Overall	*Bivariate regression analysis*					*Multiple regression analysis*				
	Mean (SD)	B	SE	t	β	*p*-value	B	SE	t	β	*p*-value
**Age**	12.74 (2.59)	0.03	0.01	3.16	0.07	0.002	-0.01	0.01	-0.61	-0.02	0.545
**Gender**											
Female	12.83 (2.74)	0.16	0.12	1.32	0.03	0.188	⸻	⸻	⸻	⸻	⸻
Male	12.68 (2.47)	Ref.									
**Marital status**											
Married	13.32 (2.49)	0.90	0.12	7.47	0.17	<0.001	0.95	0.15	6.20	0.18	**<0.001**
Divorced	14 (0)	1.58	1.05	1.51	0.03	0.130	0.36	0.97	0.37	0.01	0.709
Unmarried	12.42 (2.6)	Ref.					Ref.				
**Education**											
College	13.27 (2.86)	0.31	0.25	1.27	0.05	0.203	0.36	0.24	1.53	0.06	0.126
Bachelor	12.72 (2.52)	-0.24	0.23	-1.04	-0.05	0.298	-0.04	0.22	-0.17	-0.01	0.862
Master or above	11.82 (2.6)	-1.14	0.27	-4.29	-0.15	<0.001	-1.60	0.26	-6.21	-0.21	**<0.001**
School or below	12.96 (1.6)	Ref.					Ref.				
**Profession**											
Student	12.49 (2.78)	1.49	0.39	3.80	0.29	<0.001	1.70	0.41	4.14	0.32	**<0.001**
Housewife	13 (1.97)	2.00	0.51	3.96	0.13	<0.001	0.77	0.54	1.44	0.05	0.151
Employee	13.58 (2.38)	2.58	0.40	6.42	0.43	<0.001	2.54	0.43	5.95	0.43	**<0.001**
Businessman	11.88 (1.51)	0.88	0.44	2.01	0.09	0.044	0.99	0.47	2.12	0.10	**0.034**
Unemployed	13.17 (2.38)	2.17	0.46	4.78	0.19	<0.001	1.23	0.47	2.62	0.11	**0.009**
Day laborer	11 (0)	Ref.					Ref.				
**Socioeconomic status**											
Lower-middle class	12.25 (2.38)	-0.42	0.36	-1.15	-0.06	0.249	0.98	0.38	2.61	0.14	**0.009**
Middle class	13.29 (2.55)	0.63	0.34	1.82	0.12	0.068	2.01	0.35	5.79	0.38	**<0.001**
Higher-middle class	12.02 (2.57)	-0.65	0.36	-1.80	-0.10	0.072	0.98	0.37	2.67	0.15	**0.008**
Rich	9.73 (0.97)	-2.94	0.45	-6.48	-0.21	<0.001	-1.72	0.44	-3.86	-0.12	**<0.001**
Poor	12.67 (1.72)	Ref.					Ref.				
**Residence**											
Semi-urban	12.88 (2.04)	-0.66	0.20	-3.31	-0.10	0.001	-0.94	0.20	-4.80	-0.14	**<0.001**
Urban	12.52 (2.63)	-1.02	0.16	-6.22	-0.19	<0.001	-0.85	0.17	-5.02	-0.16	**<0.001**
Rural	13.54 (2.85)	Ref.					Ref.				

## Discussion

Multiple Antibiotic Resistance (MAR) is expected to worsen in developing countries where infectious disease is rampant due to limited medical access and poor regulation. Furthermore, community-based studies on antibiotic knowledge and attitudes are scarce, and those that have been conducted have revealed poor knowledge, attitudes and practices [9].

The purpose of this study was to determine the general population’s knowledge, attitudes, and practices regarding antibiotic use in Bangladesh. The current study found some superficiality in antibiotic knowledge, which was especially noticeable among the participants. Almost 96% participants were well known about the effectiveness of antibiotics against bacterial infections, whereas, almost 60% participants were unaware about ineffectiveness of antibiotics against viral infections. In a study in Romania, 62.65% participants considered that antibiotics are effective against bacterial infections [45] and 65.1% of respondents in Al-Ahsa, Saudi Arabia believed that antibiotics are used for the treatment of viral infection [43]. Only 35% of respondents in Kosovo answered correctly that antibiotics cannot kill viruses, whereas 42.5% gave wrong answer and 22% was unsure about the answer [46]. 73.12% of respondents stated that antibiotics could be used to treat viral infections in a study of Indonesia [47]. In our study, about 90% participants knew about antibiotic resistance, 80% were known about the adverse effects of antibiotics on normal flora where almost 93% considered that antibiotics must be bought from dispensary with prescription. This indicates overall almost 73% proper knowledge about antibiotics and their use, whereas, a study in UAE revealed that 59% participants had proper knowledge regarding antibiotic uses [38]. On the other hand, only 38.8% and 17.1% of the respondents had proper knowledge in a study of Bhutan and a tertiary care hospital in Nepal respectively [40, 48]. Almost 47% of respondents in Kuwait had low knowledge regarding antibiotic uses [41]. The findings of the present study demonstrate that the knowledge of the participants regarding antibiotic resistance and importance of prescription in purchasing antibiotics is quite high with respect to other studies. Majority of participants have information only focuses on antibacterial effects of antibiotics. Whereas, about two-thirds of people were unaware about the ineffectiveness of antibiotics against any viral infections. This implies that participants of this study might not have any lucid, comprehensive clinical and pharmacological information of antibiotics. The very low level of knowledge about ineffectiveness of antibiotics in treating any viral infections also may in turn increase the risk of antibiotic resistance.

In this study most of the participants were educated and they had knowledge about antibiotics and their use. Marital status, profession, education level and residence were significantly associated with knowledge about antibiotic use (**Table 3**).

It was found that unmarried people had proper knowledge reference to married people and those who had higher education level (college, bachelor or master degree) had significantly better knowledge than people having education level of school or below. Several studies also showed that better knowledge regarding antibiotic use is associated with a high level of education [41, 49–52] It was also significant that students, employee, businessman and housewife had better knowledge reference to day labor (**Table 3**). Similarly a study showed that 91.4% Nigerian university students had proper knowledge about antibiotics and their uses [53]. Another study also showed proper knowledge among South Indian medical students [54]. An existing study also indicated proper knowledge among university students in China [37]. Other research from many nations have described the lack of knowledge among persons with lower levels of education [13, 50, 55–58]. On the other hand, poor to moderate knowledge were observed in a study conducted in Pakistan among rural communities [44]. In this study it was also found that semi-urban and urban people had significantly better knowledge reference to rural communities (**Table 3**) which was similar to the existing study in Pakistan [44]. Lower mean score of knowledge regarding antibiotic use was found in respondents living in rural area than urban area in a study of Trinidad and Tobago [39].

The data from our research, as well as the data from other similar studies, reveal that higher educational qualifications may lead to a better understanding of the impact of improper antibiotic use. However, to ensure proper antibiotic usage and combat antibiotic resistance, the persistence of health education and awareness about antibiotic usage is necessary.

Though unmarried people had better knowledge about antibiotic use reference to married people, married people were found significantly in positive attitudes reference to unmarried people in our study. Similarly, a study in Saudi Arabia also revealed that married people had lower attitude score than unmarried people, whereas married people had better knowledge than unmarried people [49]. People of education level of college and master had positive attitudes towards antibiotic use reference to people of school or below level in our study. A study in China also indicated positive attitudes towards antibiotic use in people with higher education level [37]. Another study in Saudi Arabia revealed that people of college or above had better mean score of attitudes towards antibiotic use reference to people of school or below [49]. Profession was found to be significantly associated with attitudes towards antibiotic use. Students, housewife, employee, businessman were significantly had positive attitudes towards antibiotics reference to day laborer (**Table 4**). This study indicated that 94.14% participants were agreed to the necessity of establishment of a course on ‘rational use of antibiotics. The response rate in a study in China [37] was 63.7%, which indicated more positive attitudes among the participants in Bangladesh than in China [37]. The average attitude score was 76% in UAE [38]. Another study in Bhutan showed that 51% respondents had favorable attitude towards antibiotic use and AMR [40]. Our study showed that 92.91% participants believed that there is an abuse of antibiotics which was consistent with the study in UAE [38] and in China [37] with the percentage of 91.3% and 90.1% accordingly.

These results also indicate that unmarried and people with higher educational qualifications may possess substantial knowledge and positive attitudes towards the proper utilization of antibiotic. The possible reason might be due to their better access to the information, and improved understanding regarding antibiotics and bacterial resistance. Therefore, it may also cause to increase health awareness among them and resulting in more positive attitudes towards antibiotic use.

From this study it was found that only 57.91% participants were found to practice antibiotic use properly, whereas 73.22% and 92.67% were found to had proper knowledge and positive attitudes towards antibiotics accordingly. Though participants in this study were found to be less careful in practices of antibiotic use, the score of good practices was higher than a study of UAE (45%) which was conducted on students only [38].

Eventually the score of good practices was lower in a study of tertiary medical care in Nepal (17.1%) [48], whereas score of good practices was higher in a study among the veterinarians and para-veterinarians in Bhutan (77%) [40].

In a study among consumers in Pakistan showed that 66.7% respondents didn’t follow the actual dosage regimen [44]. In our study, it was found that married people were significantly found more careful in good practice of antibiotic uses reference to unmarried people. Similarly, a study in Saudi Arabia also showed that married people were more careful in practice of antibiotic use than unmarried people [49]. Our results clearly suggest that married people are more responsible and vigilant about the health conditions of their family members and therefore exhibit good practice about antibiotic usage. Highly educated people such as Masters or above were found in good practice of antibiotic uses than the others in this study. A study in Saudi Arabia also showed that people with high level of education had good practice rather than people with low level of education [49]. Semi-urban and urban people were more careful to use antibiotics reference to rural people. Middle class and rich socio-economic condition were significantly found to use antibiotics properly than others. Students and Employees were more careful in good practice of antibiotic uses than people of other professions (**Table 5**). The findings clearly exhibit that previously, these people might have had satisfactory outcome while treating infections with antibiotics. Moreover, satisfactory clinical outcomes, and proper information about antibiotic use and antibiotic resistance may lead to good practices of antibiotic usage.

Most serious scenario in practice of antibiotic use is being less disciplined in completing antibiotic treatment which may be a major cause of increasing antibiotic resistance. Most of the people often do not complete their course when they feel better. Though this study showed that 52.18% respondents accepted that they complete the antibiotic course properly, this percentage does not shows a good practice towards antibiotic use among the people in Bangladesh. Several studies also revealed that people were less disciplined in completing antibiotic treatment. For example, 55.1% respondents in Western Saudi Arabia [59], 51.5% of Lebanese people [51], and 42.1% of Hong Kong respondents [55] stopped their course of antibiotics when their symptoms were improved. All these studies showed similar results as our study. But best practice in completing antibiotic course properly was reported in Sweden [57], where only 5% of respondents discontinued their antibiotic treatment when their symptoms were improved. These results are indicative of adherence to the dosage regimen of antibiotics to completely get rid of their infections. The majority of people in our study, as well as several studies conducted in different countries, also adhered to their treatment with antibiotics. They might have an accurate and proper understanding about antibiotic usage and its resistance, which ultimately reflects as good practices of antibiotics among them.

Moreover, the present study showed that the respondents had proper knowledge (73.22%) and positive attitudes (92.67%) towards antibiotic use but they were less careful in practice (57.91%). This might indicate an alert of increasing antibiotic resistance, one of the most concerning issues of modern world.

## Limitations

This study contains a number of limitations that require considered. Firstly, this study employed a cross-sectional design, which precludes the establishment of causal inferences. Consequently, a longitudinal study would eliminate this barrier to understanding potential causal links. Secondly, this study has generated the information of knowledge of people on antibiotic use but it failed to concluded the source of knowledge is to be assessed in the future. Thirdly, this study also failed to demonstrate the antibiotic resistance pattern among those people who were involved in misuse of antibiotics such as buying antibiotics without prescription and were careless about to complete the course of antibiotics. Moreover, though the study included the higher number participants, most of the participants were students with a high education level. This might be cause of higher knowledge level on antibiotic use. Lastly, for comparison data between male and female, there was quite incomparable data (1112 male and 835 female) as lower number of female willingly participated in the study compared to male. Finally, it should be recognized that respondents from specific disciplines, such as those with backgrounds in medical and allied sciences, may already possess prior knowledge and attitudes regarding antibiotics. The potential bias stemming from this inherent familiarity will be duly acknowledged and thoughtfully taken into account during our analysis.

## Conclusions

The results of this study supported that highly educated people might possess substantial knowledge, and have better understanding about the impact of antibiotics and exhibit positive attitudes towards the proper utilization of antibiotics. Therefore, proper utilization of antibiotics is actually signified the existence of good practices of antibiotics. The findings of the research indicate that participants with higher educational background had acquired better knowledge regarding antibiotic resistance, adverse effects, and proper understanding of antibiotic effectiveness, which in turn had a positive impact on both attitudes and practices of antibiotics. Furthermore, this study also revealed that unmarried cognizant people, and those from upper economic status tend to exhibit positive attitudes towards good practices of antibiotic usage. The present survey also demonstrated that married people were more responsible, vigilant about antibiotics and exhibited positive attitudes and better practices. To combat the spread of antibiotic misuse and its consequences, we propose the establishment of a special course on rational antibiotic use that focuses on people’s practices toward antibiotic use rather than the advancement of knowledge and attitudes alone.

## Supporting information

S1 File Study instrument–Questionnaire. (DOCX)

S2 File Dataset of KAP. (XLSX)
