# Supplementary material for: Knowledge, attitudes, and practices regarding antibiotic use in Bangladesh: Findings from a cross-sectional study
Source: PLoS One. 2024 Feb 12;19(2):e0297653. doi: 10.1371/journal.pone.0297653 (PMC10861050; doi:10.1371/journal.pone.0297653)
Supplement: S1 File — (DOCX) [file pone.0297653.s001.docx]

**Knowledge, Attitude & Practice of Antibiotics Usage Among People of Bangladesh: A survey-based cross-sectional study**

1. Do you want to participate in our study?

Yes

No

**Category – 1: Demographic Information**

1. **Age:** __________________________________________________________________
2. **District:** _______________________________________________________________
3. **Where are you currently living?**

Town/City

Village

Sub-town

Slum

1. **Gender:**

Male

Female

Prefer not to say

1. **Profession:**

Student

Housewife

Employee

Businessman

Day laborer

Unemployed

1. **Educational qualification:**

School or below

College

Bachelor

Masters or above

1. **Marital Status:**

Unmarried

Married

Divorced/ Divorcee

Widow/Widower

1. **Economic Status:**

Rich

Higher Middle Class

Middle Class

Lower-middle class

Poor

**Category – 2: Knowledge of antibiotic usage**

1. Can antibiotics cure bacterial infections?

Yes

No

1. Can antibiotics cure viral infections?

Yes

No

1. Do you think the use of antibiotics will speed up the recovery of cold, cough?

Yes

No

1. Have you heard of antibiotics resistance?

Yes

No

1. Do you think frequent use of antibiotics will decrease the efficacy of treatment when using the antibiotic again?

Yes

No

1. Do you think antibiotics may kill the normal flora of your body?

Yes

No

1. Do you think that all bacteria are sensitive to all antibiotics?

Yes

No

1. Do you think antibiotics must be bought from a dispensary without a prescription?

Yes

No

1. Do you think antibiotics are more effective than paracetamol to cure fever quickly?

Yes

No

**Category – 3: Attitude toward the antibiotic usage**

1. There is abuse of antibiotics at present

Agree

Disagree

1. Antibiotic resistance has become a problem in Bangladesh

Agree

Disagree

1. Abuse of antibiotics has become the main cause leading to bacterial resistance

Agree

Disagree

1. Antibiotic resistance can affect you and your family’s health

Agree

Disagree

1. Necessary to get more education about antibiotics

Agree

Disagree

1. Need to establish a course on ‘rational use of antibiotics’ in educational institutions

Agree

Disagree

1. Necessary to carry out large-scale ‘antibiotics campaign’ promotion

Agree

Disagree

1. Necessary to stop selling antibiotics without prescription

Agree

Disagree

1. Necessary to highlight the side effects of antibiotic abuse

Agree

Disagree

**Category – 4: Practices of antibiotic usage**

1. Use antibiotics when having fever (temperature lower than 38.5°C or 101.3°F)

Always

Sometimes

Never

1. Use antibiotics when having common cold

Always

Sometimes

Never

1. Use of antibiotics of having acute bronchitis

Always

Sometimes

Never

1. Use antibiotics of having coughing yellow/green sputum

Always

Sometimes

Never

1. Use antibiotics of having sore throat

Always

Sometimes

Never

1. Use antibiotics of having cough with fever

Always

Sometimes

Never

1. Use antibiotics of coughing up white sputum

Always

Sometimes

Never

1. Use antibiotics of having cough lasting 2 weeks or more

Always

Sometimes

Never

1. Buy antibiotics from dispensary without prescription

Always

Sometimes

Never

1. Asked doctors to prescribe antibiotics when you catch a common cold

Always

Sometimes

Never

1. Complete the full course of antibiotics every time

Always

Sometimes

Never

**Thank you for your participation…**

**বাংলাদেশের মানুষের মধ্যে অ্যান্টিবায়োটিক ব্যবহারের জ্ঞান, মনোভাব এবং অনুশীলন: একটি সমীক্ষা-ভিত্তিক ক্রস-বিভাগীয় গবেষণা**

1. আপনি কি আমাদের গবেষণায় অংশগ্রহণ করতে চান?

হ্যাঁ

না

**বিভাগ – ১: অংশগ্রহণকারীর তথ্য**

1. **বয়স:** _____________________________________________________________________
2. **জেলা:** _____________________________________________________________________
3. **আপনি বর্তমানে কোথায় বসবাস করেন?**

শহরে

গ্রামে

উপ-শহরে

বস্তিতে

1. **লিঙ্গ:**

পুরুষ

নারী

বলতে ইচ্ছুক নই

1. **পেশা:**

শিক্ষার্থী

গৃহিণী

চাকুরীজীবী

ব্যবসায়ী

দিনমজুর

বেকার

1. **শিক্ষাগত যোগ্যতা:**

মাধ্যমিক অথবা তার কম

উচ্চ মাধ্যমিক

স্নাতক

স্নাতকোত্তর অথবা তার বেশি

1. **বৈবাহিক অবস্থা:**

অবিবাহিত

বিবাহিত

তালাকপ্রাপ্ত

বিধবা/বিপত্নীক

1. **অর্থনৈতিক অবস্থা:**

ধনী

উচ্চ-মধ্যবিত্ত

মধ্যবিত্ত

নিম্ন-মধ্যবিত্ত

দরিদ্র

**বিভাগ – ২: এন্টিবায়োটিক ব্যবহার বিষয়ক জ্ঞান**

1. এন্টিবায়োটিক কি ব্যাকটেরিয়া জনিত সংক্রমণ দূর করতে পারে?

হ্যাঁ

না

1. এন্টিবায়োটিক কি ভাইরাস জনিত সংক্রমণ দূর করতে পারে?

হ্যাঁ

না

1. আপনি কি মনে করেন এন্টিবায়োটিক ব্যবহারে সর্দি কাশি দ্রুত আরোগ্য হয়?

হ্যাঁ

না

1. আপনি কি এন্টিবায়োটিক প্রতিরোধের কথা শুনেছেন?

হ্যাঁ

না

1. আপনি কি মনে করেন যে অ্যান্টিবায়োটিকের ঘন ঘন ব্যবহার আবার অ্যান্টিবায়োটিক ব্যবহার করার সময় চিকিৎসার কার্যকারিতা হ্রাস করবে?

হ্যাঁ

না

1. আপনি কি মনে করেন এন্টিবায়োটিক আপনার শরীরের উপকারী অণুজীব মেরে ফেলতে পারে?

হ্যাঁ

না

1. আপনি কি মনে করেন সব ব্যাকটেরিয়া সব এন্টিবায়োটিকের প্রতি সংবেদনশীল?

হ্যাঁ

না

1. আপনি কি মনে করেন প্রেসক্রিপশন ছাড়াই ডিসপেনসারি থেকে অ্যান্টিবায়োটিক কিনতে হবে?

হ্যাঁ

না

1. আপনি কি মনে করেন দ্রুত জ্বর সাড়াতে প্যারাসিটামলের চেয়ে এন্টিবায়োটিক বেশি কার্যকর?

হ্যাঁ

না

**বিভাগ – ৩: অ্যান্টিবায়োটিক ব্যবহারের প্রতি মনোভাব**

1. বর্তমানে এন্টিবায়োটিকের অপব্যবহার হয়

সম্মত

সম্মত নই

1. বাংলাদেশে এন্টিবায়োটিক প্রতিরোধ একটি সমস্যা হয়ে দাঁড়িয়েছে

সম্মত

সম্মত নই

1. এন্টিবায়োটিকের অপব্যবহার ব্যাকটেরিয়ার এন্টিবায়োটিক প্রতিরোধের প্রধান কারণ হয়ে দাঁড়িয়েছে

সম্মত

সম্মত নই

1. এন্টিবায়োটিক প্রতিরোধ আপনার এবং আপনার পরিবারের স্বাস্থ্যের উপর প্রভাব ফেলতে পারে

সম্মত

সম্মত নই

1. এন্টিবায়োটিক সম্পর্কে আরো শিক্ষা দেওয়া প্রয়োজন

সম্মত

সম্মত নই

1. শিক্ষাপ্রতিষ্ঠানে ‘এন্টিবায়োটিকের যৌক্তিক ব্যবহার’ বিষয়ক একটি কোর্স চালু করা প্রয়োজন

সম্মত

সম্মত নই

1. বড় মাপের ‘এন্টিবায়োটিক ক্যাম্পেইন’ প্রচার চালানো প্রয়োজন

সম্মত

সম্মত নই

1. প্রেসক্রিপশন ছাড়া এন্টিবায়োটিক বিক্রি করা বন্ধ করা প্রয়োজন

সম্মত

সম্মত নই

1. এন্টিবায়োটিক অপব্যবহারের পার্শ্বপ্রতিক্রিয়া সবার সামনে তুলে ধরা প্রয়োজন

সম্মত

সম্মত নই

**বিভাগ – ৪: এন্টিবায়োটিক ব্যবহারের অভ্যাস**

1. জ্বর হলে এন্টিবায়োটিক ব্যবহার করি (তাপমাত্রা ৩৮.৫° সেলসিয়াস অথবা ১০১.৩° ফারেনহাইটের কম থাকলে)

সবসময়

মাঝেমাঝে

কখনই না

1. সর্দি হলে এন্টিবায়োটিক ব্যবহার করি

সবসময়

মাঝেমাঝে

কখনই না

1. ব্রংকাইটিস হলে এন্টিবায়োটিক ব্যবহার করি

সবসময়

মাঝেমাঝে

কখনই না

1. হলুদ কফ/ সবুজ থুতু এলে এন্টিবায়োটিক ব্যবহার করি

সবসময়

সবসময়

কখনই না

1. গলা ব্যথা হলে এন্টিবায়োটিক ব্যবহার করি

সবসময়

মাঝে মাঝে

কখনই না

1. জ্বরের সাথে কাশি এলে এন্টিবায়োটিক ব্যবহার করি

সবসময়

মাঝে মাঝে

কখনই না

1. কফসহ সাদা থুতু এলে এন্টিবায়োটিক ব্যবহার করি

সবসময়

মাঝে মাঝে

কখনই না

1. ২ সপ্তাহ বা তার বেশি সময় ধরে কাশি হলে এন্টিবায়োটিক ব্যবহার করি

সবসময়

মাঝে মাঝে

কখনই না

1. ডিসপেনসারি থেকে প্রেসক্রিপশন ছাড়া এন্টিবায়োটিক কিনি

সবসময়

মাঝে মাঝে

কখনই না

1. সর্দি হলে ডাক্তারকে এন্টিবায়োটিক দিতে বলি

সবসময়

মাঝে মাঝে

কখনই না

1. প্রতিবার এন্টিবায়োটিকের সম্পূর্ণ কোর্স সম্পন্ন করি

সবসময়

মাঝে মাঝে

কখনই না

**অংশগ্রহণের জন্য আপনাকে ধন্যবাদ…**
